# A Time-Varying Connectivity Analysis from Distributed EEG Sources: A Simulation Study

**DOI:** 10.1007/s10548-018-0621-3

**Published:** 2018-01-27

**Authors:** Eshwar G. Ghumare, Maarten Schrooten, Rik Vandenberghe, Patrick Dupont

**Affiliations:** 1 The Laboratory for Cognitive Neurology, Department of Neurosciences, KU Leuven, Leuven, Belgium; 2 The Neurology Department, University Hospitals Leuven, Leuven, Belgium

**Keywords:** Multivariate autoregressive (MVAR)modeling, Kalman filtering, Partial directed coherence (PDC), EEG source modeling, Visual spatial attention network

## Abstract

Time-varying connectivity analysis based on sources reconstructed using inverse modeling of electroencephalographic (EEG) data is important to understand the dynamic behaviour of the brain. We simulated cortical data from a visual spatial attention network with a time-varying connectivity structure, and then simulated the propagation to the scalp to obtain EEG data. Distributed EEG source modeling using sLORETA was applied. We compared different dipole (representing a source) selection strategies based on their time series in a region of interest. Next, we estimated multivariate autoregressive (MVAR) parameters using classical Kalman filter and general linear Kalman filter approaches followed by the calculation of partial directed coherence (PDC). MVAR parameters and PDC values for the selected sources were compared with the ground-truth. We found that the best strategy to extract the time series of a region of interest was to select a dipole with time series showing the highest correlation with the average time series in the region of interest. Dipole selection based on power or based on the largest singular value offer comparable alternatives. Among the different Kalman filter approaches, the use of a general linear Kalman filter was preferred to estimate PDC based connectivity except when only a small number of trials are available. In the latter case, the classical Kalman filter can be an alternative.

## Introduction

Brain function fundamentally relies on the interaction between functional units at different scales. Electrophysiological measures such as electroencephalography (EEG) and magnetoencephalography (MEG) can provide unique insight into the dynamic and directed interactions between anatomical regions, thanks to their high temporal resolution (Leistritz et al. 2016; Lopes da Silva 2013). This relies on the validity of methods and strategies used to derive time-varying directed connectivity from EEG and MEG when cortical sources are estimated (Siebenhü et al. 2016; Mahjoory et al. 2016).

The technique to map EEG data from sensor space to cortical sources is referred to as EEG source modeling. Popular approaches for distributed source modeling are the weighted minimum-norm estimate (Jeffs et al. 1987) and standardized low-resolution brain electromagnetic tomography (sLORETA) (Pascual-Marqui 2002). Both methods are widely used to study directed and time-varying EEG-based connectivity between sources (Wang et al. 2016; Simpson et al. 2011; Hassan and Wendling 2015; Plomp et al. 2016; Gao et al. 2015; Hassan et al. 2014). sLORETA is robust against noise, is less biased towards superficial sources and the solutions are very smooth. Once the sources are determined, the connectivity between these sources can be studied using a variety of methods such as Granger causality (GC) (Freiwald et al. 1999), phase synchronisation (Campbell et al. 1980) or cross-spectrum (Blackman and Tukey 1959) among the reconstructed time series (Hassan et al. 2017; Haufe and Ewald 2016). All these methods heavily depend on the accuracy of the time series in the selected sources. In the case of smooth distributed sources, the extraction of the correct representative time series is far from trivial while at the same time, this is critical for an accurate estimate of the connectivity measure (Mahjoory et al. 2016). Often, time series are averaged across the dipoles in a region of interest (ROI) which leads to an additional smoothing. An alternative is to extract the time series from a single dipole (Sohrabpour et al. 2016; Coito et al. 2016). However, when using such a strategy, it is important to evaluate the performance of different dipole selection strategies within an ROI.

Once the time series are extracted in selected dipoles, directed and time-varying connectivity between these sources can be studied to determine information processing in the human brain (Leistritz et al. 2016; Lie and Mierlo 2017; Liu et al. 2016; Mao et al. 2016; Plomp et al. 2016). Unlike functional connectivity, directed and time-varying connectivity allows to study the information flow and the timings of the interactions among brain regions to understand the basis of cognitive functions. Among the different approaches to derive directed and time-varying connectivity, multivariate autoregressive modeling (MVAR) and the concept of GC, are widely applied (Baccalá and Sameshima 2001). GC based measures give directed flow by estimating a linear causal relationship among brain regions. In this article, we focused on partial directed coherence (PDC), one of the commonly applied GC based frequency domain measures. The estimation of PDC follows the MVAR modeling of EEG time series. The estimated MVAR parameters are transformed to the frequency domain to calculate PDC values. The conventional approaches are based on stationary MVAR estimates of the data i.e. one model is estimated for the entire length of the time series. However, EEG is highly non-stationary, and stationarity will miss the dynamic interactions among brain regions. With a moving window approach, this would still require stationarity in a window and the size of the window will impose further limitations to the results. Among all time-varying MVAR estimation approaches, a Kalman filter based MVAR modeling gained wider applications in high-dimensional EEG data due to its accurate estimation of non-stationary (Milde et al. 2010; Arnold et al. 1998). Kalman filter based approaches can track transient changes in spectra of EEG data and give estimates of the MVAR model at each time point so that time-varying PDC can be calculated. A Kalman filter can be implemented in a number of ways to estimate the time-varying MVAR model.

Here we present a methodological investigation on time-varying connectivity starting from EEG source modeling. More specifically, our aim was:

1. To compare strategies for dipole selection within an ROI after source modeling.
2. To compare the performance of time-varying directed connectivity methods based on different Kalman filtering approaches to derive PDC based networks.

To perform a methodological investigation a ground truth time varying connectivity is required. Such validation is not possible with real data and simulations are inevitable and the only way to compare different methods. Simple simulations are useful to gain insight into the behaviour of a method under different conditions like SNR, but ultimately we want to apply such methods in more complex situations, and therefore the development of more realistic simulations is essential (Haufe and Ewald 2016). We used simulated EEG data with a known ground truth time-varying directed connectivity model. A preliminary version of this work with a simple model consisting of three nodes has been reported in (Ghumare et al. 2015).

## Methods

### Time-Varying Connectivity

We first describe the theoretical formulation of time-varying directed connectivity based on GC starting from time series in a set of sources.

For the discrete time series $y \in R^{\ m \times N}$ measured in *m* channels with *N* samples, the time-varying MVAR process is described as:

1 $$\begin{aligned} y(n)= \sum _{k=1}^{p} A_{k}(n) \ y(n-k) + e(n) \end{aligned}$$

where *n* being the n-th time bin of the *N* samples, *p* is the model order, $A_{k}(n) \in R^{\ m \times m}$ is the matrix of the time-varying MVAR model parameters at time bin *n* for delay *k*, $k =1, 2,\ldots , { p}$ and *e*(*n*) is a vector of multivariate zero-mean uncorrelated white noise.

Partial directed coherence is a full multivariate spectral measure based on the concept of GC (Baccalá and Sameshima 2001), used to determine the directed influences between a pair of time series in sources *i* and *j* with the influence of the remaining time series removed. Using time-varying MVAR parameters, we can obtain time-varying PDC values from source *j* to source *i* calculated as a function of frequency and time:

2 $$\begin{aligned} \pi _{ij}(f,n) = \frac{\bar{A}_{ij}(f,n)}{\sqrt{\sum \limits _{r=1}^{m} \bar{A}_{rj}(f,n) \ \bar{A}_{rj}^{H}(f,n)}} , \quad \sum _i{|{\pi _{ij}(f,n)}|^2} = 1 \end{aligned}$$

in which the superscript *H* stands for the Hermitian transpose and

3 $$\begin{aligned} \bar{A}(f,n) = I - \sum _{k=1}^{p} A_{k}(n) e^{-i2\pi fk} \end{aligned}$$

where *f* is the normalized frequency in the interval [− .5, .5]. We used the squared values of PDC i.e. ${|{\pi _{ij}(f,t)}|^2}$ as measure of connectivity. Squared values of PDC were shown to provide superior accuracy and sensitivity compared to PDC (Astolfi et al. 2006).

### Time Varying MVAR Model Estimation Using Kalman Filtering

The application of the Kalman filtering algorithm to MVAR modeling is based on a linear state-space representation of the signal. A linear state-space model consists of two joined linear equations:

the state equation

4 $$\begin{aligned} \tilde{A}_{p}(n+1) = \tilde{A}_{p}(n) + v(n) \end{aligned}$$

and a measurement equation

5 $$\begin{aligned} y(n) = H_{p}(n) \tilde{A}_p(n) + e(n) \end{aligned}$$

The state equation relates state $\tilde{A}_{p}(n)$ of MVAR parameters at time bin *n* to the state or MVAR estimates at time bin $n+1$ with $v(n)\sim \mathscr {N}(0, V(n))$, the state white noise process and $H_{p}(n)$ is a matrix with the *p* past data points of the measurement. The time-varying MVAR parameters $\tilde{A}_{p}(n)$ are related to the parameters $A_k(n)$ (see Appendices 1 and 2).

The MVAR parameters $\tilde{A}_{p}(n)$ are estimated using Kalman filtering recursion equations. There are mainly two different implementations of Kalman filtering to perform time-varying MVAR modeling: the classical Kalman filter (CKF) (Arnold et al. 1998) or the general linear Kalman filter (GLKF) (Milde et al. 2010). The former is implemented for a single trial while the latter has an implementation which takes into account multi-trial data and which is not a straightforward extension of the CKF, i.e. it does not reduce to the CKF if one would consider single trial data as a special case of multi-trial data. The details of the CKF and the general linear Kalman filter are given in Appendices 1 and 2 respectively.

For multi-trial EEG/ERP data, we can use the following strategies to estimate time-varying PDC:

1. PDC values are calculated from the averaged single trial MVAR estimates using the classical Kalman filter (CKF-1) (Tang et al. 2013);
2. PDC values are calculated by averaging (across trials) single trial estimates of the PDC values (CKF-2) calculated from MVAR estimates using the classical Kalman filter (Eftaxias and Sanei 2013; Omidvarnia et al. 2014);
3. PDC values are calculated from MVAR estimates obtained using the general linear Kalman filter.

Previously, we have shown that averaging the trials before MVAR modeling will result in inaccuracies (Ghumare et al. 2015) and therefore, this approach will not be one of our strategies.

### Simulated Ground Truth Data

To compare the different strategies, we used simulated data with a ground-truth model at the level of cortical sources.

**Fig. 1:**
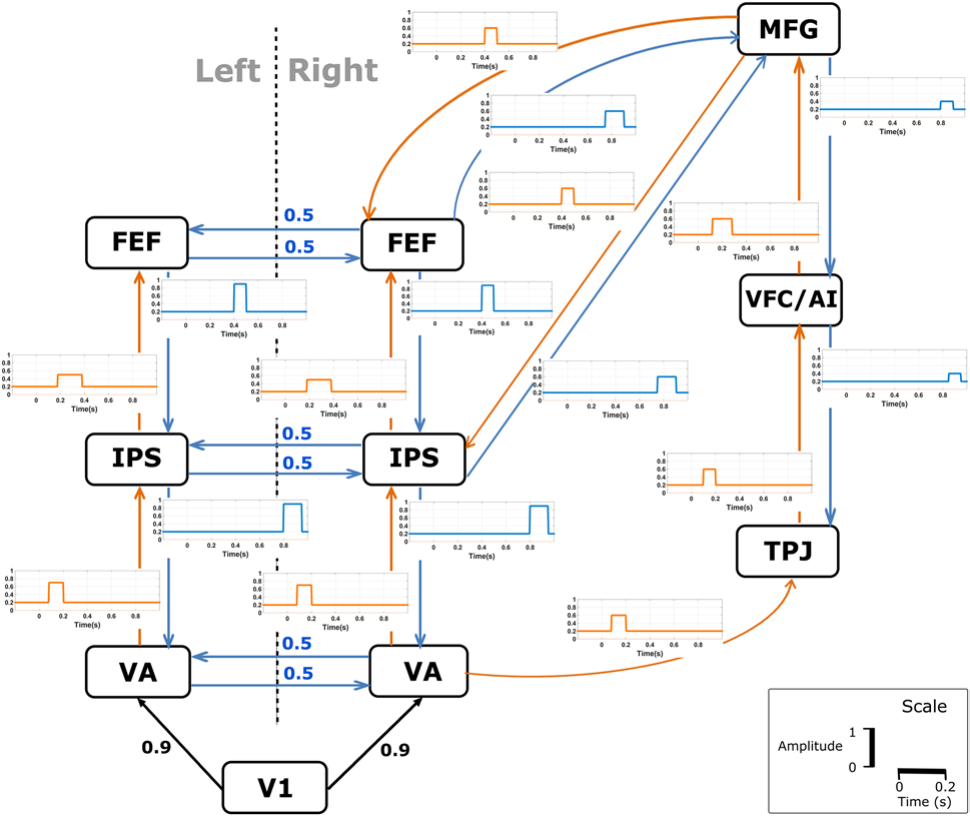
The simulated visual spatial attention model consisting of an input area (V1), two visual areas (VA), the intraparietal sulcus (IPS), the frontal eye fields (FEF), the temporoparietal junction (TPJ), the anterior insula in the ventral frontal cortex (VFC/AI) and the middle frontal gyrus (MFG). The model was taken from (Corbetta et al. 2008) with some minor modifications: connections between FEF, IPS, and MFG were slightly adapted, and the visual input region was added. The arrows indicate directed interactions consisting of a stimulus-driven control (orange), top-down control (blue) and the visual input signal (black). Bidirectional interhemispheric connections were modeled as stationary with a strength of 0.5. The time-varying MVAR connectivity was imposed based on the timings of the significant effects observed in different regions as described in (Simpson et al. 2011) and (Vossel et al. 2014) and are shown by the figures next to each directed connection. These time-varying connections were added on top of the stationary connection in which the latter had a strength of 0.2. The time lag for MVAR parameters for the connection in blue and orange was chosen as 16 ms and for black as 4 ms. The exact onset of the directional time-varying interactions, its amplitudes and duration as well as the time lag were chosen arbitrarily

The simulated data consisted of a realistic large-scale model of the visual spatial attention system with a complex time-varying and directed connectivity structure. The simulated directed connectivity model is shown in Fig. 1. The connectivity model was based on (Corbetta et al. 2008) and the time-varying information was based on the timings of the significant effects observed in different regions as described in (Simpson et al. 2011) and (Vossel et al. 2014). The timings were specified for the presentation of a central cue in a visual spatial attention experiment.

To mimic the visual input to the cortical areas, the input signal was obtained from a source estimated from real EEG data acquired during a visuospatial attention experiment in a healthy control. In this experiment, the trials started with a central cue presented for 200 ms indicating the direction of attention to the left. After a delay phase of 300 ms from cue offset, a grating in the left hemifield was shown in combination with a central fixation cross. We estimated the cortical sources using distributed source modeling (Pascual-Marqui 2002) with the head model derived from a high-resolution anatomical MRI of that subject. To extract the visual input, a signal from 200ms before cue onset until 500 ms after cue onset (the end of the delay phase) was extracted from a source in primary visual cortex (V1) and it was resampled with a sampling frequency of 256 Hz. The source in V1 was located at MNI coordinates (6.3, − 82.3, − 3.7) corresponding to a central position in the visual field according to retinotopic mapping studies (Dougherty et al. 2003). Because the timings of the effects were described in (Simpson et al. 2011; Vossel et al. 2014) for 1000 ms after stimulus onset, we had to generate a new input signal for a longer duration. This was done as follow: (1) we estimated stationary autoregressive parameters from the original input signal using the ARFIT package (Schneider and Neumaier 2001) and (2) we used the estimated parameters to simulate the new input signal for a duration of 1000 ms after stimulus onset. This signal was used as input for the model (Fig. 1). The model order for the stationary autoregressive model was determined using Schwarz’s Bayesian Criterion (SBC) and was found to equal 10. The model order was further validated based on the comparison of the power spectrum of the signal using a non-parametric Welch and parametric Burg method (van Mierlo et al. 2013). Furthermore, the frequency spectrum of the simulated signal confirmed the presence of a standard 1/*f* function with a peak in the alpha band (8–12 Hz) as in a real EEG frequency spectrum.

The cortical signals of the ground truth model in the other regions were generated using the time-varying MVAR model shown in Fig. 1. The sampling frequency was set to 256 Hz. The noise amplitude was adjusted at each time point to achieve a constant SNR level of 20. The model was constructed iteratively in time to generate single trial data. We repeated the procedure to obtain 100 trials to mimic multiple trial data. The time series in each trial was generated for a duration of 1200 ms in which the first 200 ms were considered baseline.

Next, we associated the multi-trial ground truth time series to the cortical sources (dipoles) which were located on the cortical surface using the default anatomy (Colin27) in Brainstorm and which were closest to the Montreal Neurological Institute (MNI) coordinates described in Table 1 based on the Euclidean distance. The MNI coordinates from Table 1 were derived from previous studies on visuospatial attention (Gillebert et al. 2013; Simpson et al. 2011).

**Table 1:** MNI coordinates of the cortical ground truth sources

Region	x	y	z
Primary visual cortex (V1)	6.3	− 82.3	− 3.7
Right visual area (R VA)	15	− 71	5
Right intraparietal sulcus (IPS R)	42	− 42	48
Right frontal eye fields (FEF R)	38	− 6	56
Right temporoparietal junction (TPJ R)	66	− 48	20
Right ventral frontal cortex/anterior insula (VFC/AI R)	39	0	39
Right middle frontal gyrus (MFG R)	47	38	29
Left visual area (VA L)	− 14	− 81	9
Left intraparietal sulcus (IPS L)	− 44	− 57	48
Left frontal eye fields (FEF L)	− 43	− 7	52

### Simulated Scalp EEG Data

Using the data in the ten dipoles, we simulated EEG measurements in 256 electrodes derived from the ANT Neuro sensors available in Brainstorm. The forward matrix *G* was estimated with the symmetric boundary element method (Gramfort et al. 2010) implemented in Brainstorm. This model consisted of three layers: skin, skull, and brain [including cerebrospinal fluid (CSF)] with relative values of the conductivities set as 1, 1/80 and 1 S/m respectively (Qin et al. 2010; Ahrens et al. 2012). The conductivity ratio between scalp and skull was set to 1/80 which was the default value in Brainstorm. However, others have argued that this ratio is rather between 1/20 and 1/10 (Oostendorp et al. 2000; Lai et al. 2005). By performing the simulations using the value 1/80, the problems caused by the conductivity of the skull become more pronounced and it could be considered as a worst case scenario.

The brain sources were limited to the cortical surface with 15,002 vertex points with a dipole orientation orthogonal to the cortical surface. For each time bin n, we can calculate the surface EEG signal *D(n)* from the forward matrix *G* and the signal *S(n)* in all dipoles:

6 $$\begin{aligned} D(n) = G \cdot S(n) + e(n) \end{aligned}$$

in which *e*(*n*) is white noise mimicking measurement noise.

We applied an overall scale factor for *S* to obtain a peak amplitude in the EEG data in the same range as realistic measurements. EEG scalp signals were calculated with an average reference.

We adapted the variance of the white noise to generate trials at a specific SNR level. SNR was defined as the ratio of the average power in the EEG recordings (over all trials, time and EEG electrodes) to the power of the white noise added (Leistritz et al. 2013). Final datasets consisted of 100 trials per SNR level.

### Source Modeling of Simulated EEG Data

We followed a realistic approach by performing a data-driven distributed source modeling using Brainstorm.

The noise covariance of the EEG data required for the inverse estimation was calculated using the baseline period of 200 ms of all simulated trials. Off-diagonal elements of the noise covariance were discarded to model uncorrelated measurement noise. The parameter $\lambda$ used in Brainstorm is required for the regularization of the ill-posed problem. $\lambda$ is related to the level of noise present in the measurements and is calculated as $\lambda = 1/\text {SNR}^2$ in which SNR represents the signal to noise ratio (Bradley et al. 2016). For each dataset, the simulated SNR of the scalp EEG data was used to calculate $\lambda$. To regularize the noise covariance matrix we used the default setting of 0.1 in Brainstorm. During the source estimation, the orientations of the dipoles were constrained to be normal to the cortical surface. A shared inversion kernel for all the trials was determined using sLORETA implemented in Brainstorm (Pascual-Marqui 2002). The estimated shared kernel was applied to each trial of EEG data to obtain the corresponding cortical signal in each dipole position in each of the 15002 vertices.

### Regions of Interest and Dipole Selection

Using the scout menu in Brainstorm, we created a-priori regions of interest (ROIs) on the cortical surface around the location of the ground-truth dipoles (Table 1). Each ROI consisted of 40–50 vertex points (corresponding to an area of 10 cm^2^) and was defined using the position of the ground-truth dipole as as seed. Within each ROI we selected a single dipole from the distributed sources obtained during source modeling. Extraction of a single time series in a ROI better overcomes the problem of smoothness of the inverse solution compared to the averaged time course of all dipoles within that ROI (Rueda-Delgado et al. 2017). The set of time series of the selected dipoles was used to perform the connectivity analysis.

We compared a number of strategies to select a single, representative dipole within an ROI. We used two different types of strategies for the dipole selection: (1) strategies in which we used the ground truth information and (2) strategies in which we use a data driven approach (as we would do in a real experiment).

#### Dipole Selection Using Ground Truth Information

In these approaches, we used the ground truth knowledge to select the representative dipole in each ROI. The following strategies were used:

1. The ground truth dipole was selected (GT1).
2. The dipole in the ROI with the highest correlation between the time series in that dipole and the ground truth time series was selected (GT2) (Babiloni et al. 2004).

#### Data Driven Methods for the Dipole Selection

In these approaches, we selected a dipole without the knowledge of the ground truth data. Before selecting the dipole, we have to determine the dominant direction of the dipoles in an ROI followed by sign flipping the dipoles with opposite direction (Hassan et al. 2017). The reason for this approach is that the inverse solution obtained by sLORETA is based on minimum norm estimates and the sign of these minimum norm estimates depends on the dipole direction. In some of the strategies, we make use of the resolution matrix of the inverse solution which is the product of the inverse kernel and the forward matrix. In the ideal case, the resolution matrix will be the identity when sources are perfectly separated. However, this is never the case because of the ill-posed nature of the problem. The selection strategies we have evaluated were:

1. The dipole with the highest correlation between the time series in that dipole and the averaged time series across all dipoles in the ROI. Such a dipole could best represent the regional fluctuations in the signal (DD1).
2. The dipole with the highest power (i.e. the mean squared amplitude) (DD2) (Rueda-Delgado et al. 2017).
3. The dipole showing the highest correspondence with the largest singular value based on a row singular vector (Sohrabpour et al. 2016). Such a dipole can best explain the variability in the ROI (DD3).
4. The dipole with the resolution index closest to 1 in the ROI (DD4) (Stenroos and Hauk 2013; Hauk et al. 2011). The resolution index is the diagonal value of the resolution matrix, and 1 indicates that the sources are optimally resolved.
5. The dipole with the highest cross-talk function (CTF) index in the regions (DD5). This index was defined as the ratio of the mean outflow CTF (the sum of the column elements of the resolution matrix) and the mean inflow CTF (the sum of the row elements of the resolution matrix). A “strong” dipole will have more outflow CTF due to the smooth solution and a minimal inflow CTF indicating its closeness to the ideal solution (Farahibozorg et al. 2017; Hauk et al. 2011).

We used different parameters to evaluate the performance of the different dipole selection strategies:

1. The Euclidean distance and the surface based distance between the position of the selected dipole and the ground truth position.
2. The Pearson correlation coefficient between the time series in the selected dipole and the ground-truth time series.
3. The mean squared error (MSE) between the linear fit of the time series in the selected dipole and the ground-truth time series.

These parameters were estimated for different SNR levels.

### Evaluation of Different Kalman Filtering Approaches

The Kalman filtering approaches were applied to the time series extracted from the dipoles selected after source modeling to determine time-varying MVAR parameters followed by time-varying PDC estimation.

The SNR of the surface EEG and the number of trials were chosen as factors to vary when evaluating the different Kalman filter approaches. We used SNR = [1, 3, 5, 10] and number of trials = [3, 5, 10, 20, 40, 60, 80, 100]. For each setting, we used 100 noise realizations and each noise realization was obtained by repeating the entire process starting from the simulated EEG surface recordings.

The update constant $U_C$ was set to 0.02 (Astolfi et al. 2008; Leistritz et al. 2013) and we used a fixed model order of eight in all subsequent analyses. This model order was obtained by fitting a stationary MVAR model to the ground truth data using the ARFIT algorithm from the time series analysis toolbox (Schlögl 2002; Schneider and Neumaier 2001) and by applying the SBC criterion because it is least affected by the presence of noise (Porcaro et al. 2009).

Based on the theoretical time-varying MVAR parameters shown in Fig. 1, we constructed the theoretical time-varying PDC values (Astolfi et al. 2008). For the calculation of PDC, we limited our analysis to the frequency window 1–40 Hz based on the spectral power of the ground truth source data. We used two figures of merit to compare the performance of each method for MVAR model parameters and PDC separately. The figures of merit were estimated per factor level and per noise realization. Since it may make a difference whether we look at existing or non-existing connections in the ground truth model, we performed a separate analysis for both types of connections.

The first figure of merit was the MSE between the theoretical and estimated time-varying MVAR parameters:

7 $$\begin{aligned} \text {MSE}_{\text {MVAR}} = \mathbf {E} [( \tilde{A}_p(n)^{\text {estimated}}- \tilde{A}_p(n)^{\text {theoretical}})^2] \end{aligned}$$

where *p* is the model order, *n* is the time bin.

The MSE between the theoretical and estimated time-varying PDC values was used as another figure of merit:

8 $$\begin{aligned} \text {MSE}_{\text {PDC}} = \mathbf {E} [({|\pi _{ij}(f,n)^{\text {estimated}} |}^2- {|\pi _{ij}(f,n)^{\text {theoretical}}|}^2)^2] \end{aligned}$$

where *i* and *j* refers to a pair of ROIs, *f* is the frequency bin, *n* is the time bin. In Eq. 8, the diagonal elements are excluded due to the column normalization properties of PDC.

We performed repeated-measures ANOVAs for $\text {MSE}_{\text {MVAR}}$ and $\text {MSE}_{\text {PDC}}$ to compare the three Kalman filter based approaches. A Greenhouse-Geisser correction for sphericity was used. The posthoc analysis was performed using Scheffé’s method. The statistical significance was set at* p* < 0.05 Bonferroni corrected for the number of pairwise tests performed.

## Results

### Dipole Selection to Extract ROI Time Series

**Fig. 2:**
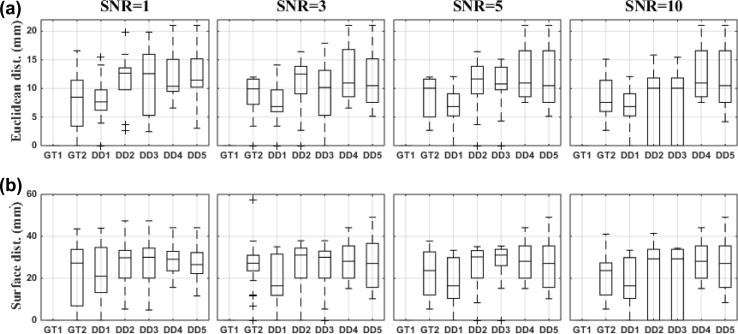
The performance parameters for the dipole selection strategies at different levels of SNR. Box-and-Whisker plots across all regions and 100 noise realizations are shown. **a** Euclidean distance in mm from the ground truth location. **b** Surface distance from the ground truth location along the cortex

**Fig. 3:**
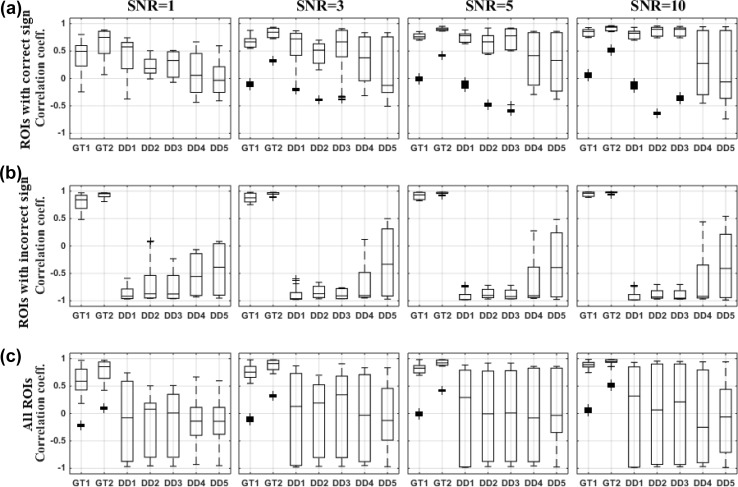
The correlation coefficient with corresponding ground truth time series for the dipole selection strategies at different levels of SNR. Box-and-Whisker plots across all regions and 100 noise realizations are shown. **a** ROIs with the correct sign of the dominant direction compared to the ground truth direction. **b** ROIs with an incorrect sign of the dominant direction compared to the ground truth direction. **c** Overall results

**Fig. 4:**
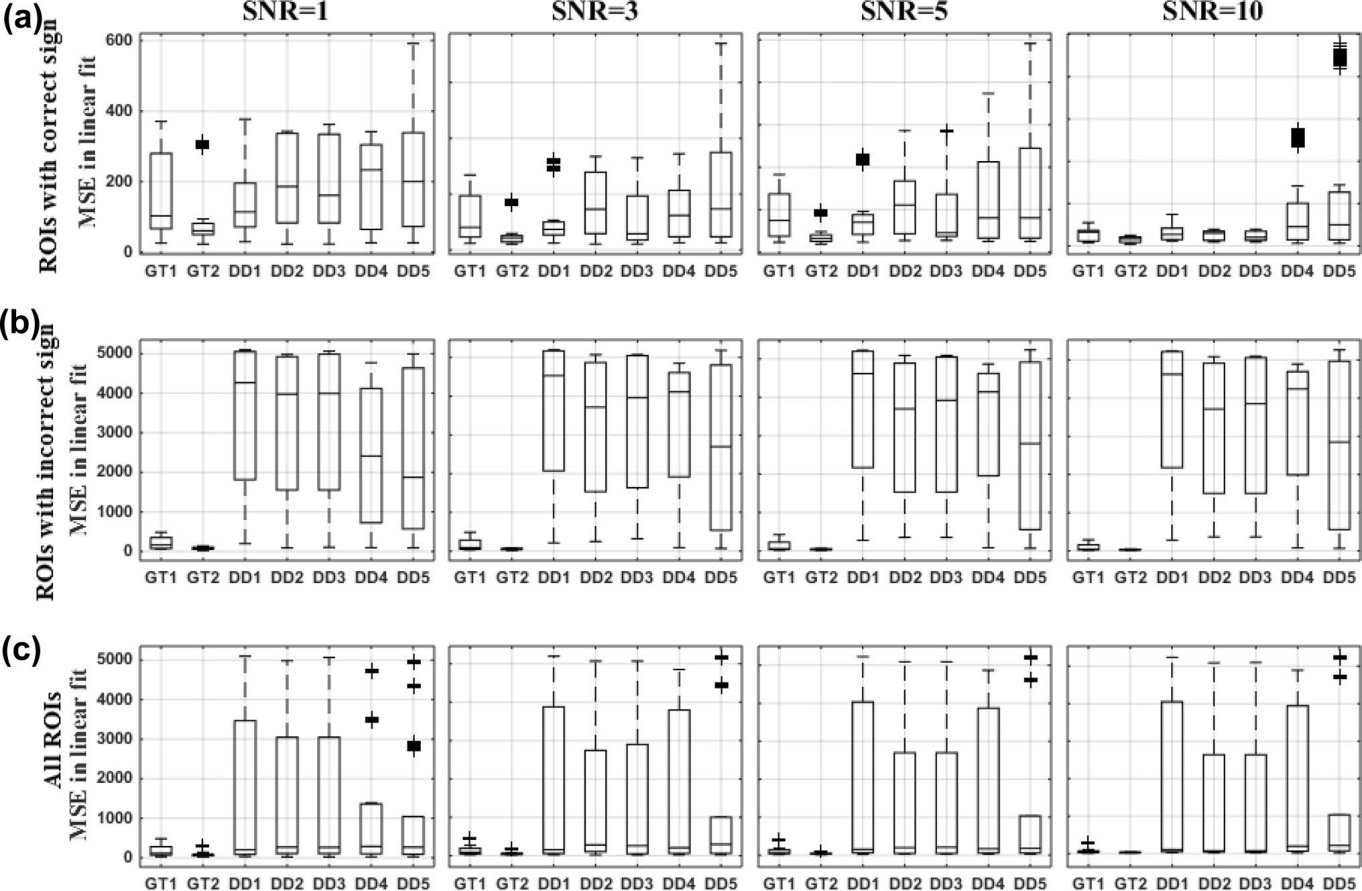
Mean square error (MSE) after linear fitting with ground truth time series for the dipole selection strategies at different levels of SNR. Box-and-Whisker plots across all regions and 100 noise realizations are shown. **a** ROIs with the correct sign of the dominant direction compared to the ground truth direction. **b** ROIs with an incorrect sign of the dominant direction compared to the ground truth direction. **c** Overall results

We compared different dipole selection strategies using various performance parameters in two different scenarios: (1) when the ground truth is known and (2) using data-driven methods (Figs. 2, 3 and 4).

For data driven dipole selection methods, we selected the dominant direction of the dipoles in the ROI without the ground truth knowledge similar to a real experiment. As a result, in four ROIs the sign of the dominant direction was opposite to the ground truth direction. The dipole selection itself was not affected but only the sign of the dipole time series. As a result, the localization error was not affected but the correlation with the ground truth time series and the MSE with these time series was strongly affected. Therefore, we showed additional results for ROIS in which the extracted time series had a correct or incorrect sign separately (panels a and b in Figs. 3, 4).

Compared to the other data-driven strategies, DD1 showed the lowest localization error both using the Euclidean distance (Fig. 2a) and using the surface distance (Fig. 2b) . The localization error improved with increasing levels of SNR.

Looking at the correlation between the selected time series and the ground truth time series, we observe that in ROIs with the correct sign for the dominant direction, the dipole showing the highest correlation with the ROI averaged time series (DD1) also showed a high correlation with the ground truth time series (Fig. 3a). The performance of this strategy for this criterion was comparable to the ground truth based dipole selection strategies. For DD2 (power based selection) and DD3 (based on SVD), the performance improved with increasing levels of SNR and was comparable to DD1 when SNR=10 (Fig. 3a). The strategies based on resolution index (DD4) and the CTF index (DD5) showed a lower performance (Fig. 3a). For ROIs with an incorrect sign for the dominant direction, we observed almost an opposite pattern: using DD1, DD2 and DD3 lead to strong negative correlations and using DD4 and DD5 was found to be superior (Fig. 3b). Based on the overall results for all ROIs, we observed comparable performances of all the approaches for this criterion (Fig. 3c). However, based on the median of the data, we considered DD1, DD2 and DD3 as superior compared to DD4 and DD5. Improvement in the performance of using DD2 and DD3 with increasing SNR was consistently observed. At SNR=10, DD2 and DD3 showed results comparable to DD1.

Looking at the MSE between the linear fit of the selected time series and the ground truth time series, DD1 also showed the best performance for this criterion for ROIs with the correct sign of the dominant direction (Fig. 4a). DD1 showed minimal variation across regions and noise realizations compared to all other data-driven methods. Overall the error reduced with increasing levels of SNR, and this was the case for all the dipole selection strategies (Fig. 4a). Similar to the results for the correlation coefficient, all methods showed for the ROIs with an incorrect sign of the dominant direction a large variability as well as a higher error compared to the ROIs with a correct sign of the dominant direction (Fig. 4b). The overall results also indicated a large variability (Fig. 4b) and DD4 and DD5 can be considered the best for this criterion.

In the remainder, we will show the performance of the Kalman filtering approaches for dipole selection strategy DD1, DD2 and DD3 since they can be considered the best taking all criteria into account. For comparison, we also used the ground truth based selection strategy GT2 since this is also a correlation based strategy.

### Performance of Kalman Filtering Approaches

The Kalman filtering approaches were compared at different levels of SNR and number of trials. The figures of merit were calculated separately for time-varying MVAR parameter estimates and PDC values. To distinguish between the performance for existing versus non-existing connections in the ground truth network, we applied the figures of merits separately for both types of connections.

#### Errors in Existing Connections

The figures of merit for the existing connections indicate the sensitivity to capture the time-varying connectivity in the underlying brain network.

**Fig. 5:**
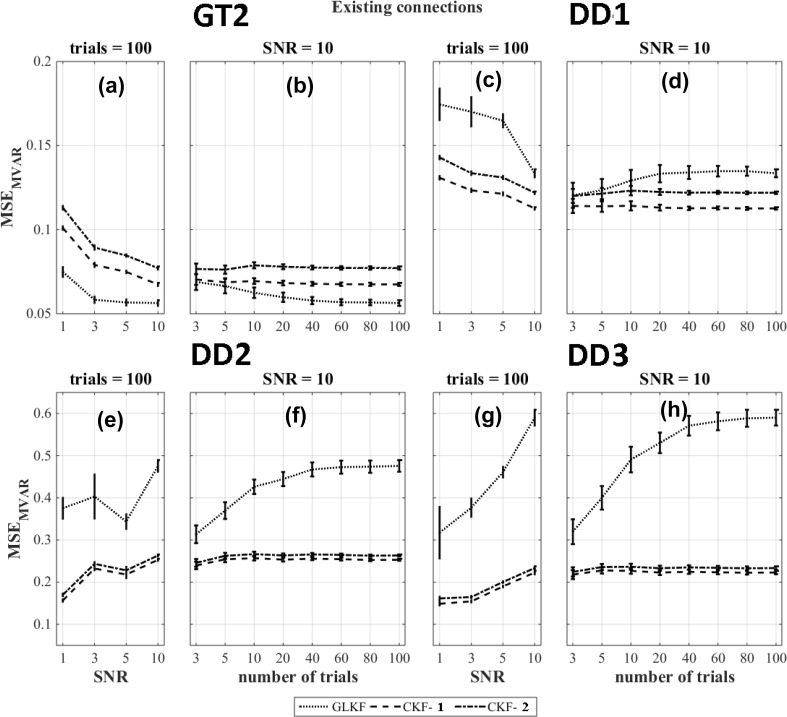
$\text {MSE}_{\text {MVAR}}$ for different Kalman filtering approaches at various levels of SNR and number of trials for existing model connections and using the ground truth based dipole selection GT2 and the data driven dipole selections DD1, DD2 and DD3

The results of $\text {MSE}_{\text {MVAR}}$ for existing connections and the ground truth based dipole selection (GT2) and data driven dipole selection strategies DD1, DD2 and DD3 are shown in Fig. 5a–h. For $\text {MSE}_{\text {MVAR}}$, the use of the general linear Kalman filter outperformed the other approaches ($p < 0.05$) when using the ground truth based dipole selection (GT2) (Fig. 5a–b). The figures of merits calculated for the data-driven dipole selection methods (DD1, DD2 and DD3) are shown in Fig. 5c–h. Averaging of the MVAR estimates after using the classical Kalman filter (CKF-1) outperforms the other methods at all levels of SNR and number of trials while GLKF showed the worst performance. We also observed in some cases an increase in error when the number of trials increased or when SNR increased. When looking at individual MVAR plots, this was caused by the sign flip in four of the time series as a result of the wrong dominant direction within the corresponding ROI.

**Fig. 6:**
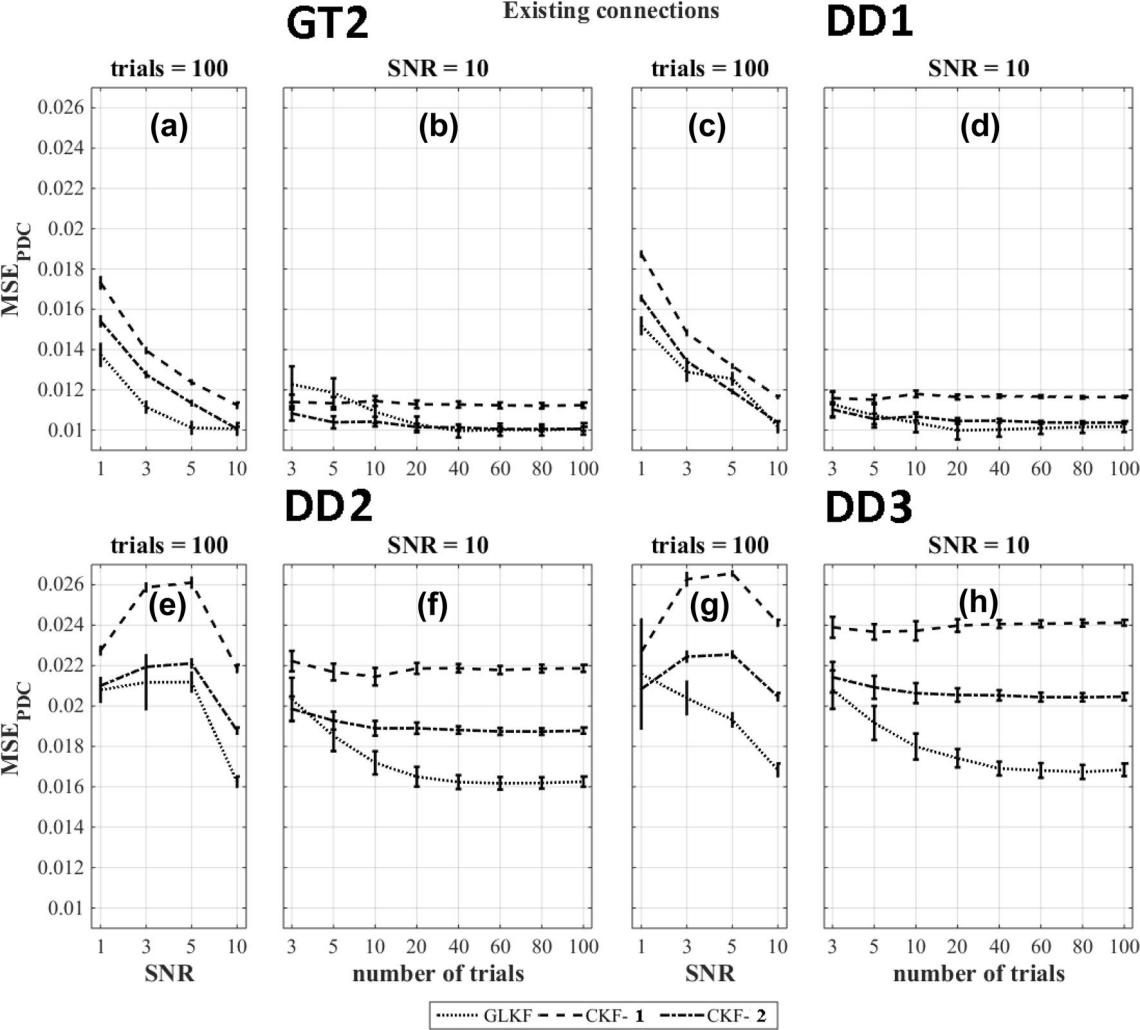
$\text {MSE}_{\text {PDC}}$ for different Kalman filtering approaches at various levels of SNR and number of trials for existing model connections and using the ground truth based dipole selection GT2 and the data driven dipole selections DD1, DD2 and DD3

For $\text {MSE}_{\text {PDC}}$, the use of the general linear Kalman filter outperformed the other approaches when the dipole was selected based on the ground truth based strategy (GT2) except when the number of trials was ≤ 20 at SNR=10 in which case averaging PDC values across single trial estimates of the PDC values using the CKF is the best method (CKF-2) (Fig. 6a–b). For data driven dipole selection methods, the use of the general linear Kalman filter outperformed ($p < 0.05$) the other methods in most situations (Fig. 6c–h). When using DD1, GLKF and CKF-2 gave comparable errors in PDC values (Fig. 6c–d).

#### Errors in Non-existing Connections

The figures of merit for the non-existing connections is an indication for detection of false positive connections.

**Fig. 7:**
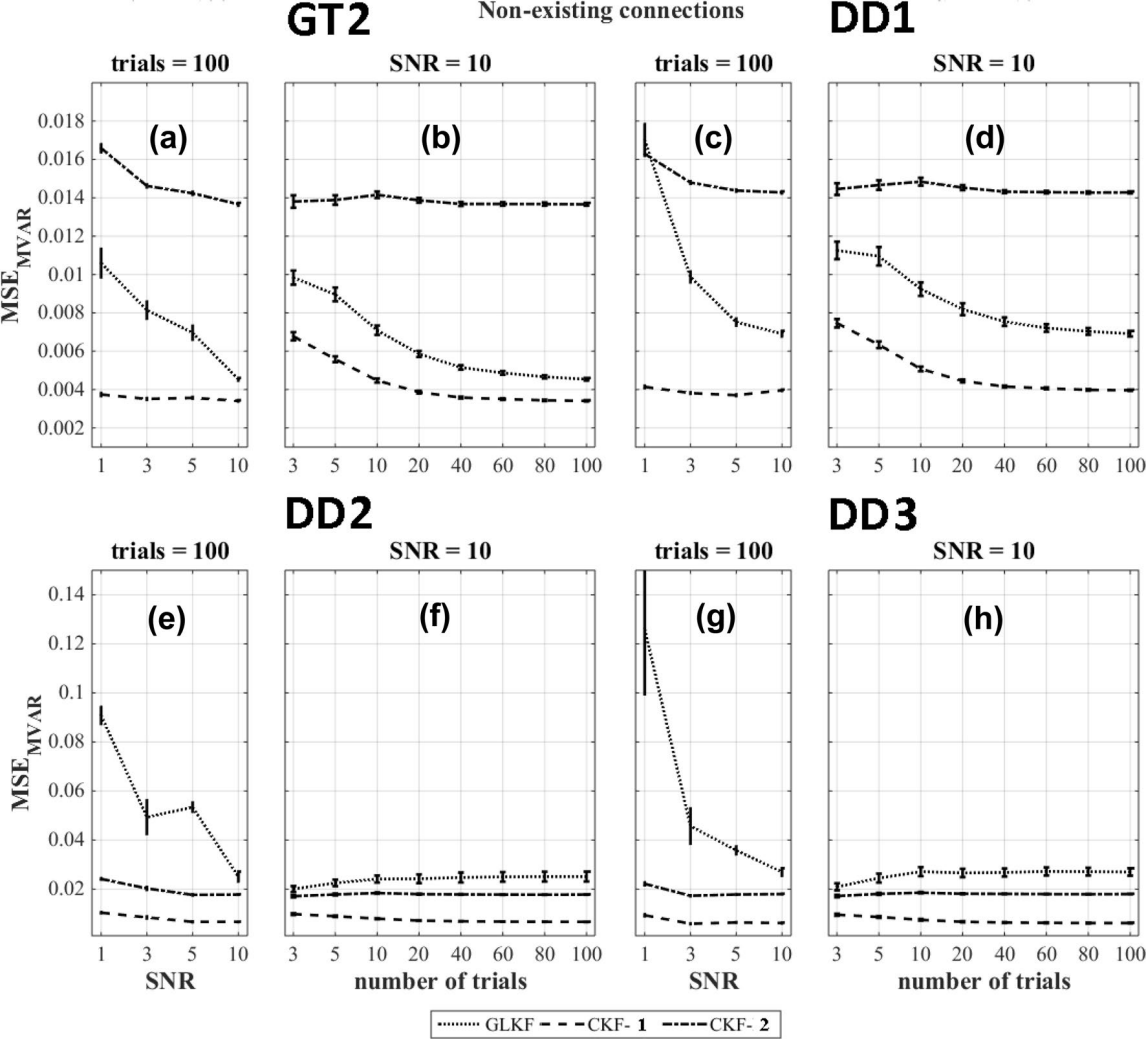
$\text {MSE}_{\text {MVAR}}$ for different Kalman filtering approaches at various levels of SNR and number of trials for non-existing connections of the model and using the ground truth based dipole selection GT2 and the data driven dipole selections DD1, DD2 and DD3

The results of the figures of merit $\text {MSE}_{\text {MVAR}}$ for the non-existing connections and the ground truth based dipole selection (GT2) and data driven dipole selection strategies DD1, DD2 and DD3 are shown in Fig. 7a–h. The results indicate that averaging of the MVAR estimates after using the classical Kalman filter (CKF-1) outperforms the other methods ($p < 0.05$).

**Fig. 8:**
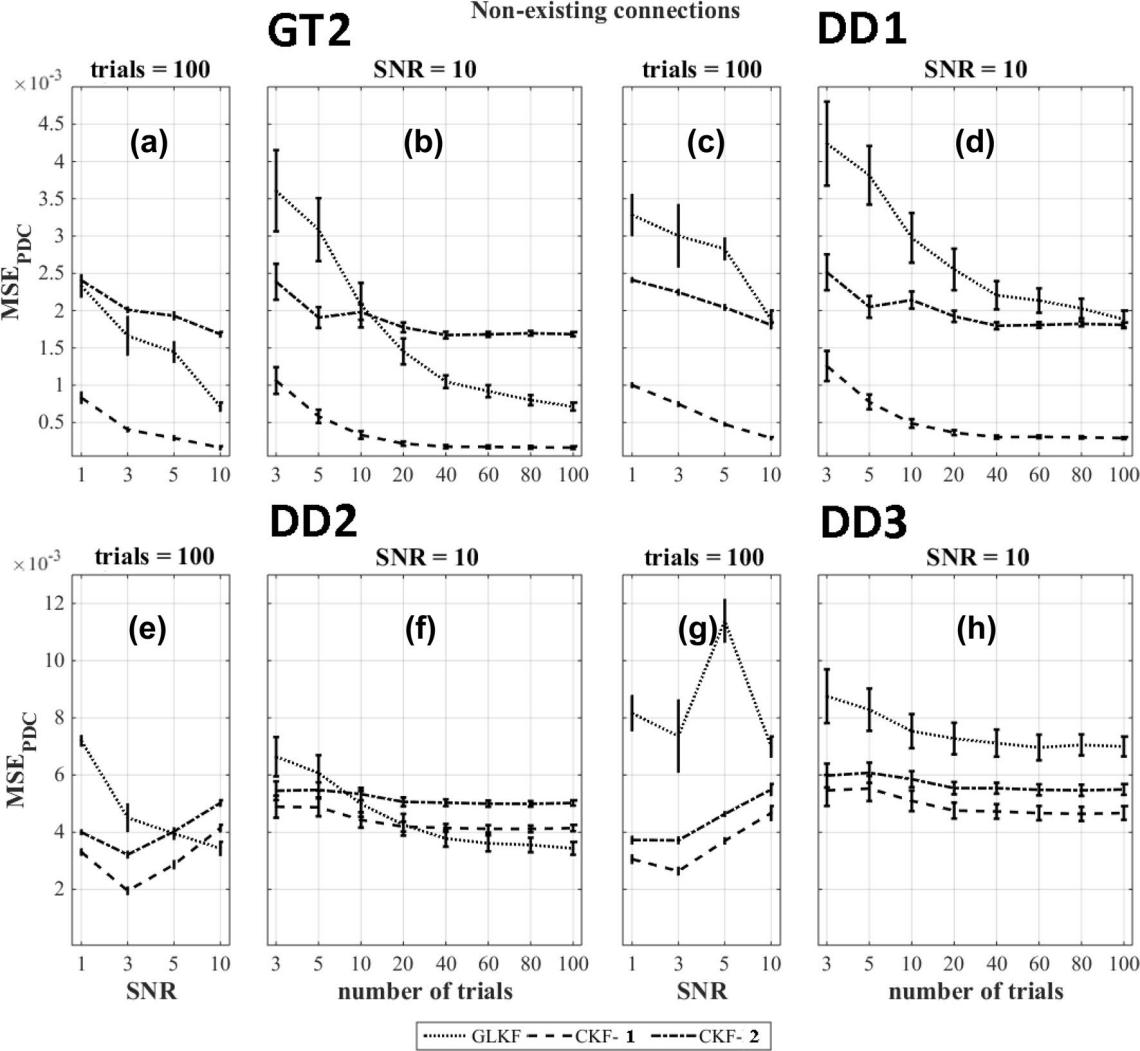
$\text {MSE}_{\text {PDC}}$ for different Kalman filtering approaches at various levels of SNR and number of trials for non-existing connections of the model and using the ground truth based dipole selection GT2 and the data driven dipole selections DD1, DD2 and DD3

The results of the figures of merit $\text {MSE}_{\text {PDC}}$ for the non-existing connections and the ground truth based dipole selection (GT2) and data driven dipole selection strategies DD1 , DD2 and DD3 are shown in Fig. 8a–h. Similar to $\text {MSE}_{\text {MVAR}}$, the results indicate that the classical Kalman filter with averaging of the MVAR estimates (CKF-1) outperforms the other methods ($p < 0.05$).

#### Overall Performances

Overall the performance depends on the ratio of existing and non-existing connections as well as on their actual errors. In our case, for $\text {MSE}_{\text {MVAR}}$, the use of the general linear Kalman filter outperformed all other approaches for the ground truth based dipole selection. For data-driven dipole selection methods DD1, DD2 and DD3, this was the case for averaging of the MVAR estimates after using the classical Kalman filter (CKF-1).

For $\text {MSE}_{\text {PDC}}$, the use of the general linear Kalman filter outperformed the other methods for all the dipole selection approaches (GT2, DD1, DD2 and DD3).

Interesting to note is that among the data-driven dipole selection methods, $\text {MSE}_{\text {MVAR}}$ and $\text {MSE}_{\text {PDC}}$ was lowest for DD1.

## Discussion

The pipeline to derive time-varying connectivity from EEG data can be divided into three stages: (1) estimation of cortical sources (source modeling); (2) ROI selection and time series extractions; and (3) estimating time-varying connectivity. There is abundant literature available about source modeling, and therefore we investigated the remaining two stages of the pipeline that required further attention. We used simulated data with a ground-truth time-varying connectivity applied to regions involved in the visual spatial attention system. We studied two aspects: first, we compared strategies to select representative dipoles from which the time series could be used in the connectivity analysis and second, we compared the performance of different Kalman filtering approaches in deriving time-varying PDC based connectivity.

Some of the earlier work on time varying connectivity (Wilke et al. 2008) focused on the application of the classical Kalman filter to compare the adaptive and the stationary directed transfer function. In contrast, in this work, we focused on the comparison between the CKF and the more recent general linear Kalman filter for the case of multi-trial data and their impact on the estimation of the MVAR model parameters and the PDC values along with the comparison of dipole selection methods. Previously, the methodological investigations on time-varying connectivity approaches were often based on simulated EEG data with only a few network nodes with a simple time-varying structure and without EEG cortical source estimation (Wilke et al. 2008; Astolfi et al. 2008; Leistritz et al. 2013). However, in this study, we took it a step further and used a model based on the visual spatial attention system. This model of the attention system (Corbetta et al. 2008) has been highly influential and had the regions sparsely distributed all over the cortical surfaces at various depths. Such a configuration allowed to compare the different approaches under more realistic circumstances with respect to other simulations in which only a few regions with a simple time-varying connectivity structure are used.

In our model, we simulated 23 directed connections from 90 possible connections. The simulated directed connections allowed the modeling of feed-forward and feedback mechanisms of the interaction between areas as is the case in a real brain network (Corbetta et al. 2008). Furthermore, time-varying influences on top of baseline connectivity mimic cognitive processes and flow of information between regions. However, the exact choices of the time-varying values of MVAR parameters were arbitrary but consistent with the timings described in (Simpson et al. 2011; Vossel et al. 2014) specified for the presentation of a central cue in a visual spatial attention experiment.

The source modeling included in the simulation pipeline is essential for the comparison of the performance because this is what we do in a real experiment. We used a realistic head model in combination with the symmetric boundary element method and constrained the orientation of the sources orthogonal to the cortical surface. However, we did not want to include the effect of the creation of the head model on the dipole selection and the comparison of the performance of different Kalman filtering approaches, and therefore we made the same choices for the head model while simulating the surface EEG data from the ground truth model. The distributed source estimation using minimum norm is giving the network that best matched the ground-truth (Hassan et al. 2017). Among minimum norm algorithms, sLORETA is widely applied due to its standardization applied to the estimates to reduce the error in depth localization. However, the performance of sLORETA to uncover multiple source configurations with different strengths and cortical depths is still under investigation (Becker et al. 2016; Dümpelmann et al. 2012) although the approach is a promising candidate and performs well as compared to other linear approaches for source localizations (Dümpelmann et al. 2012; Wagner et al. 2003).

The smoothed distributed sources, obtained using sLORETA, result in mixing of sources due to cross-talk and impose a primary challenge to estimate the connectivity. A reliable estimation of the true connectivity is possible if the shape and fluctuations of the source’s time series are well estimated. Often the time series of an ROI is obtained by averaging the time series across dipoles within that ROI (Hassan et al. 2017), However, this would further worsen the problem for GC and phase based connectivity measures (Ghumare et al. 2015; Makeig 2002). To overcome this problem, choosing a single representative dipole is recommended (Rueda-Delgado et al. 2017; Sohrabpour et al. 2016; Coito et al. 2016). We compared a number of strategies for dipole selection. A large correlation indicates a strong matching of the shape of the ground-truth and the time series in the selected dipole (Stenroos and Hauk 2013; Babiloni et al. 2003, 2004 ). Another criteria often applied is mean squared error between times series in estimated and true sources. However, compared to conventional criteria, we used MSE between the time series in the true source and a linear fit of time series in the estimated source. Due to the ill-posed nature, the strength of estimated sources is underestimated compared to the strength of true sources with a factor of about $10^{-3}$ (Stenroos and Hauk 2013). MSE calculated by the direct comparison between estimated sources and true sources would lead to a dipole selection with higher amplitude but with less similarity in signal fluctuations. However, the fluctuations are essential to extract the time-frequency characteristics and connectivity. Our approach of linear fitting of estimated time series to the ground truth time series ensured that the selected dipole time series has a similar shape as the true source. Note that we did not perform the connectivity calculations with scaled data but performed it with the unscaled estimated time series.

For the sources estimated with constrained orientations (normal to the cortex), the sign of the estimated time series can be an issue. A strategy that is often used, is to determine the dominant direction of the ROI based on the scalar product of the orientations followed by a sign flip of the dipole time series that are not in the dominant direction (Hassan et al. 2017). For the regions used in this study, we found four ROIs in which the sign of the dominant direction was opposite compared to the ground truth. This has no impact on the dipole selected but it has an impact on the sign of the time series which will be used in the connectivity analysis. When we evaluated the dipole selection methods, we found that the methods based on highest correlation (DD1), highest power (DD2) or using SVD (DD3) performed comparatively well. Dipole selection based on the resolution matrix showed the worst performance. This is caused by selecting a dipole with (almost) no signal since such dipoles can also have a resolution index of 1 or can have a high cross-talk function index when the dipole is surrounded by very low signal dipoles resulting in a low denominator (inflow cross-talk function).

The comparison of different Kalman filtering approaches to derive time-varying PDC was performed using four dipole selection strategies (one which was based on knowledge of the ground-truth and three purely data-driven methods). The figures of merit calculated for time-varying MVAR indicated how well the simulated model is extracted. We found that averaging of the MVAR estimates after using the classical Kalman filter (CKF-1) gave the best result for all data driven dipole selection strategies. In this analysis we included four time series with the wrong sign because the dominant direction in the corresponding ROIs was sign flipped compared to the ground truth. As a result, we observed a decline in the performance with increasing levels of SNR or number of trials. But if we are interested in directed connectivity, we are using the MVAR parameters to calculate the PDC values and these were not much affected by the sign flip. However, the accuracy of MVAR parameters is usually considered important for the generalization of the results to other measures (Sameshima et al. 2015).

Overall, based on the $\text {MSE}_{\text {PDC}}$ results, the best Kalman filtering approach depends on the number of trials and the SNR in the data. However, some clear trends can be observed. For existing connections, a higher number of trials is required for the approach in which we use the general linear Kalman filter in order to outperform the other strategies (Ghumare et al. 2015). There should be sufficient data compared to the number of estimated parameters depending on the model order and the number of time series (Schlögl and Supp 2006). For the case of non-existing connections, the noise in the data can often lead to false positive connections. In that case, the best performance was obtained when averaging MVAR estimates across trials (CKF-1), and this is caused by the improved SNR while averaging. The use of the general linear Kalman filter showed poor performance for the non-existing connections. However, the error was much lower compared to the existing connections and therefore, this method was overall the best one in most cases. However, if we look at the ground truth based method GT2, we speculate that the general linear Kalman filter eventually would outperform CKF-1 in case of non-existing connections if we would have included a higher number of trials. For the data-driven approaches, this is a bit more complicated because in that case the influence of the incorrect sign of some of the time series is also playing a role.

Interestingly, based on $\text {MSE}_{\text {MVAR}}$ and $\text {MSE}_{\text {PDC}}$, we observed a lower error using the ground truth based (GT2) and data driven DD1 dipole selection method compared to the other data driven methods DD2 and DD3. This also supports our idea that for time-varying connectivity studies, the dipole selection should not be based on amplitude but on the fluctuations in the signal which are more relevant in that case.

There are a number of limitations in our analysis. Firstly, often in source simulation studies, random noise is added to dipoles besides the ground truth dipoles to mimic the background brain activity (Haufe and Ewald 2016; Babiloni et al. 2003, 2004). However, there are several noise configurations possible. In reality, each noise configuration can lead to slightly different results, and none can be considered as the best choice. In our analysis, noise in the ground truth sources is required due to the intrinsic property of MVAR approaches being a white noise process. Therefore, we added only a small amount of noise to the ground truth sources (SNR = 20) to mimic the background noise. Furthermore, we added noise at the level of the scalp in various amounts. Therefore, we did not add any noise in the dipoles besides the ground truth dipoles to model background brain activity but rather considered it negligible. Secondly, in the dipole selection strategies, the results were based on a surface ROI. We have performed additional analyses with a surface ROI of smaller size compared to the original analysis and a spherical ROI of 1 cm radius. These additional analyses showed similar results in the dipole selection strategies.

## Conclusions

We compared approaches for single dipole based extraction of time series from the inversely reconstructed EEG sources in regions of interest. We showed that a single dipole can be selected to represent the time series based on the highest correlation with the averaged time series in the ROI. The dipole selected based on the highest power or based on a singular value decomposition are good alternatives. The comparison of different approaches based on Kalman filtering to estimate time-varying PDC showed that the best approach is based on the use of the general linear Kalman filtering in case of existing connections whereas the CKF with trial averaged MVAR model estimates is the best approach for non-existing connections. Based on the overall performance, the general linear Kalman filter is the best choice.

## Appendix 1: Classical Kalman Filter (CKF)

CKF can be estimated using a single trial of the data. Let $\tilde{A}_p(n)$ and $H_p(n)$ be given as:

9 $$\begin{aligned} \tilde{A}_p(n) = \begin{pmatrix} vec[A_{1}^\mathsf {T}(n)]^\mathsf {T}\\ \vdots \\ vec[A_{p}^\mathsf {T}(n)]^\mathsf {T} \end{pmatrix} \in R^{\ mmp \times 1} \end{aligned}$$

in which *vec* means the vectorization of the matrix $A_k(n)$ by selecting row by row and

10 $$\begin{aligned} H_p(n) = I_{\ m \times m} \otimes \begin{pmatrix} y^\mathsf {T}(n-1)\\ \vdots \\ y^\mathsf {T}(n-p) \end{pmatrix} \in R^{\ m \times mmp} \end{aligned}$$

where $\otimes$ denotes the Kronecker product of matrices. For *m* channel measurement of *N* samples, $y \in R^{\ m \times N}$, CKF is defined as follows:

For ${\text {n}}=[1,\ldots ,{\text {p}}]$, initialize the time-varying MVAR parameters $\tilde{A}_p(n) = 0$, the a-posteriori error covariance matrix $P(n) = I$ and the measurement error covariance $W(n) = I$. For each time bin *n*, apply the Kalman filtering recursion equations (Arnold et al. 1998):

11 $$\begin{aligned}&\text {Find the measurement error: } e(n) = y(n) - H_p(n) \tilde{A}_{p}(n-1) \end{aligned}$$

12 $$\begin{aligned}&\text {Update the measurement error covariance: } \nonumber \\&W(n) = (1-U_C)W(n-1) + U_C (e(n) \ e(n)^\mathsf {T}) \end{aligned}$$

13 $$\begin{aligned}&\text {Calculate the residual covariance: } \nonumber \\&X(n) = [H_p(n) P(n-1)H_p(n)^\mathsf {T} + \ W(n)]^{-1} \end{aligned}$$

14 $$\begin{aligned}&\text {Calculate the Kalman gain: } K_{G}(n) = P(n-1)H_p(n)^\mathsf {T}X(n) \end{aligned}$$

15 $$\begin{aligned}&\text {Update the MVAR estimates: } \tilde{A}_p(n) = \tilde{A}_p(n-1) + K_{G}(n)e(n) \end{aligned}$$

16 $$\begin{aligned}&\text {Calculate the state}\ \text { error covariance: } \nonumber \\&V(n) = \frac{U_C \text {trace}([I - K_{G}(n)H_p(n)]P(n-1))}{m m p}I \end{aligned}$$

17 $$\begin{aligned}&\text {Update the a-posteriori error covariance: }\nonumber \\&P(n) = [I - K_{G}(n)H_p(n)]P(n-1) + V(n) \end{aligned}$$

The Update coefficient $U_C$ ($0< U_C < 1$) controls the adaptation speed of time-varying MVAR parameters $\tilde{A}_p(n)$. CKF was implemented using the *mvaar.m* function available from the time series analysis toolbox (Schlögl 2002).

## Appendix 2: General Linear Kalman Filter (GLKF)

GLKF can estimate the time-varying MVAR model for multi-trial data. Let $\tilde{A}_p(n)$ and $H_p(n)$ be given as:

18 $$\begin{aligned} \tilde{A}_p(n) = \begin{pmatrix}A_{1}(n)\\ \vdots \\ A_{p}(n)\end{pmatrix} \in R^{\ mp \times m} \end{aligned}$$

and

19 $$\begin{aligned} H_p(n) = [O(n-1) O(n-2)\ldots O(n-p)] \end{aligned}$$

where

20 $$\begin{aligned} O(n) = \begin{pmatrix} y(1,n,1) &{} y(2,n,1) &{}\cdots &{} y(m,n,1) \\ y(1,n,2) &{} y(2,n,2) &{}\cdots &{} y(m,n,2) \\ \vdots &{} \ddots &{} \vdots &{} \vdots \\ y(1,n,K) &{} y(2,n,K) &{} \cdots &{} y(m,n,K) \end{pmatrix} \in R^{\ K \times m} \end{aligned}$$

For *m* channel measurements with number of trials K, $y \in R^{\ m \times N \times K}$, GLKF is defined as follows (Milde et al. 2010):

For n=[1,…,p], initialize the time-varying MVAR parameters as $\tilde{A}_p(n) = 0 \in R^{\ mp \times m}$, the a-posteriori error covariance matrix $P(n) = I \in R^{\ mp \times mp}$ and the measurement error covariance $W(n) = I \in R^{\ m \times m}$. For each time bin *n*, apply the Kalman filtering recursion equations (Milde et al. 2010):

21 $$\begin{aligned}&\text {Find the measurement error:} e(n) = y(n)^\mathsf {T} - H_p(n)\tilde{A}_p(n-1) \end{aligned}$$

22 $$\begin{aligned}&\text {Update the measurement error covariance: } \nonumber \\&W(n) = (1-U_C)W(n-1) + \frac{U_C\ e(n)^\mathsf {T}e(n)}{K-1} \end{aligned}$$

23 $$\begin{aligned}&\text {Calculate the residual covariance: } \nonumber \\&X(n) = [H_p(n)P (n-1)H_p(n)^\mathsf {T} + \text {trace}(W(n))I]^{-1}\end{aligned}$$

24 $$\begin{aligned}&\text {Calculate the Kalman gain: } K_{G}(n) = P (n-1)H_p(n)^\mathsf {T}X(n) \end{aligned}$$

25 $$\begin{aligned}&\text {Update the MVAR estimates: } \tilde{A}_p(n) = \tilde{A}_p(n-1) + K_{G}(n)e(n) \end{aligned}$$

26 $$\begin{aligned}&\text {Calculate the state}\ \text { error covariance: } \nonumber \\&V(n) = \frac{{U_{C} {\text {trace}}\left( {[I - K_{G} (n)H_{p} (n)]P(n - 1)} \right) }}{{mmp}}I \end{aligned}$$

27 $$\begin{aligned}&\text {Update the a-posteriori error covariance: } \nonumber \\&P(n) = [I - K_{G}(n)H_p(n)]P(n-1) + V(n)\ \end{aligned}$$

The General Linear Kalman filter was implemented in MATLAB using custom-written scripts.
